# Supplementary material for: MetaRibo-Seq measures translation in microbiomes
Source: Nat Commun. 2020 Jun 29;11:3268. doi: 10.1038/s41467-020-17081-z (PMC7324362; doi:10.1038/s41467-020-17081-z)
Supplement: Supplementary file 10 — Supplementary Data 7 [file 41467_2020_17081_MOESM10_ESM.zip › File2/Confidence_High_Taxonomy/442208_out.krona.html]

Javascript must be enabled to view this page.

members
magnitude
magnitudeUnassigned
count
unassigned
taxon
rank

442208\_out

188

superkingdom
2
188

976
phylum
186

186
200643
class

186
171549
order

family
815
186

genus
816
186

1
species
1739517

SRS104485\_contig\_number\_838

3
species
817

SRS063985\_contig\_number\_24623SRS104975\_contig\_number\_15563SRS147346\_contig\_number\_10537


SRS015890\_contig\_number\_1767
species
457391
1

1
species
1262750

SRS055966\_contig\_number\_191


SRS023583\_contig\_number\_7975
1
162156
species


SRS011084\_contig\_number\_34572SRS013158\_contig\_number\_144SRS013382\_contig\_number\_560SRS013638\_contig\_number\_14324SRS013965\_contig\_number\_40824SRS014287\_contig\_number\_17087SRS014313\_contig\_number\_1670SRS014412\_contig\_number\_17275SRS014613\_contig\_number\_244SRS014736\_contig\_number\_7577SRS014855\_contig\_number\_14932SRS014948\_contig\_number\_2688SRS015190\_contig\_number\_3109SRS015217\_contig\_number\_22506SRS015486\_contig\_number\_910SRS015663\_contig\_number\_34161SRS015694\_contig\_number\_22082SRS015782\_contig\_number\_4839SRS016495\_contig\_number\_4179SRS016753\_contig\_number\_3306SRS016897\_contig\_number\_486SRS017103\_contig\_number\_6789SRS017247\_contig\_number\_2662SRS017859\_contig\_number\_2006SRS019178\_contig\_number\_4411SRS019397\_contig\_number\_3277SRS019445\_contig\_number\_25736SRS019693\_contig\_number\_6941SRS019808\_contig\_number\_15551SRS020394\_contig\_number\_10459SRS020508\_contig\_number\_147SRS021484\_contig\_number\_22498SRS022137\_contig\_number\_17290SRS022609\_contig\_number\_31418SRS023971\_contig\_number\_1517SRS024075\_contig\_number\_9892SRS024132\_contig\_number\_21157SRS024435\_contig\_number\_33770SRS024492\_contig\_number\_12213SRS024625\_contig\_number\_9099SRS043001\_contig\_number\_536SRS043411\_contig\_number\_12698SRS043701\_contig\_number\_3401SRS045244\_contig\_number\_821SRS045826\_contig\_number\_6214SRS050026\_contig\_number\_6209SRS050422\_contig\_number\_24837SRS050941\_contig\_number\_48SRS050974\_contig\_number\_contig-100\_26.24065SRS051610\_contig\_number\_9736SRS051882\_contig\_number\_10710SRS052576\_contig\_number\_169SRS052697\_contig\_number\_1610SRS053214\_contig\_number\_13665SRS053335\_contig\_number\_15444SRS054059\_contig\_number\_5159SRS054352\_contig\_number\_1404SRS057480\_contig\_number\_10292SRS058268\_contig\_number\_2SRS058723\_contig\_number\_997SRS062701\_contig\_number\_5643SRS063127\_contig\_number\_1180SRS064645\_contig\_number\_1021SRS064973\_contig\_number\_119SRS065397\_contig\_number\_3990SRS075716\_contig\_number\_1401SRS075773\_contig\_number\_15300SRS075821\_contig\_number\_13358SRS076876\_contig\_number\_19SRS077502\_contig\_number\_16509SRS077641\_contig\_number\_18971SRS077991\_contig\_number\_contig-100\_80.23987SRS078176\_contig\_number\_15565SRS097958\_contig\_number\_6603SRS098061\_contig\_number\_10265SRS100021\_contig\_number\_7011SRS104084\_contig\_number\_12895SRS1041036\_contig\_number\_2284SRS1041091\_contig\_number\_9375SRS1041092\_contig\_number\_10248SRS1041118\_contig\_number\_15587SRS1041134\_contig\_number\_283SRS1041138\_contig\_number\_1526SRS1041142\_contig\_number\_1697SRS1041143\_contig\_number\_6392SRS1041144\_contig\_number\_1897SRS1041147\_contig\_number\_495SRS1055022\_contig\_number\_11260SRS1055049\_contig\_number\_4027SRS1055056\_contig\_number\_10020SRS142599\_contig\_number\_15132SRS142712\_contig\_number\_2481SRS142923\_contig\_number\_2157SRS143523\_contig\_number\_16560SRS143722\_contig\_number\_4221SRS143991\_contig\_number\_28575SRS144297\_contig\_number\_14831SRS144537\_contig\_number\_22666SRS144714\_contig\_number\_4845SRS145308\_contig\_number\_3923SRS147377\_contig\_number\_4780SRS147614\_contig\_number\_7688SRS148319\_contig\_number\_15548SRS148784\_contig\_number\_13454SRS148817\_contig\_number\_2210SRS892882\_contig\_number\_10063SRS893172\_contig\_number\_9230SRS893187\_contig\_number\_12967SRS893230\_contig\_number\_7668SRS893285\_contig\_number\_632SRS893295\_contig\_number\_10827SRS893383\_contig\_number\_26289
821
species
112

species
820
2

SRS050998\_contig\_number\_13150SRS074670\_contig\_number\_705


SRS017375\_contig\_number\_100
1
species
310298

371601
species
3

SRS049402\_contig\_number\_16089SRS050299\_contig\_number\_4779SRS064276\_contig\_number\_3238


SRS016056\_contig\_number\_351
1638779
species
1


SRS012273\_contig\_number\_47339SRS017433\_contig\_number\_11768SRS078665\_contig\_number\_2144SRS104400\_contig\_number\_18806SRS893380\_contig\_number\_948
5
46506
species

species
246787
7

SRS011239\_contig\_number\_12264SRS013476\_contig\_number\_40081SRS015579\_contig\_number\_7811SRS024331\_contig\_number\_16650SRS077730\_contig\_number\_17557SRS144183\_contig\_number\_29078SRS971275\_contig\_number\_11524


SRS143598\_contig\_number\_18423
1263042
species
1

4
species
2292935

SRS048060\_contig\_number\_3644SRS1055043\_contig\_number\_26583SRS1055067\_contig\_number\_4260SRS1055099\_contig\_number\_13749

species
47678
2

SRS077335\_contig\_number\_2088SRS143342\_contig\_number\_10855

18
357276
species

SRS013098\_contig\_number\_5102SRS013940\_contig\_number\_3450SRS018541\_contig\_number\_10792SRS018575\_contig\_number\_5156SRS018984\_contig\_number\_5903SRS019286\_contig\_number\_1308SRS024549\_contig\_number\_566SRS052078\_contig\_number\_1832SRS054590\_contig\_number\_304SRS056519\_contig\_number\_598SRS058770\_contig\_number\_5589SRS1041038\_contig\_number\_15204SRS1041112\_contig\_number\_1715SRS1041136\_contig\_number\_18487SRS140645\_contig\_number\_976SRS149784\_contig\_number\_20478SRS149879\_contig\_number\_21737SRS971276\_contig\_number\_31616

species
674529
1

SRS147766\_contig\_number\_395

1
species
626931

SRS017521\_contig\_number\_39247


SRS142503\_contig\_number\_20738
species
1263038
1

28116
species
4

SRS046369\_contig\_number\_5814SRS053649\_contig\_number\_12184SRS149075\_contig\_number\_11615SRS893253\_contig\_number\_1034

1
species
2292944

SRS018133\_contig\_number\_6010

2
329854
species

SRS016954\_contig\_number\_9038SRS104636\_contig\_number\_2325

2292923
species
1

SRS016381\_contig\_number\_2565

1
1263054
species

SRS1041039\_contig\_number\_20060

species
310297
1

SRS049712\_contig\_number\_15599

2292943
species
3

SRS023914\_contig\_number\_5861SRS077024\_contig\_number\_4833SRS147139\_contig\_number\_2163


SRS015794\_contig\_number\_2253SRS016840\_contig\_number\_2209SRS021219\_contig\_number\_8920SRS048981\_contig\_number\_3062SRS057717\_contig\_number\_609SRS098717\_contig\_number\_11931SRS1041090\_contig\_number\_5412
7
818
species

1
phylum
74201

203494
class
1

1
48461
order

1647988
family
1

239934
genus
1


SRS146813\_contig\_number\_26496
1
species
239935

phylum
1239
1

class
91061
1

1
186826
order

1
family
1300

1
1301
genus


SRS076821\_contig\_number\_contig-100\_971.151487
species
1305
1
